# 2166: Semantic characterization of clinical trial descriptions from ClincalTrials.gov and patient notes from MIMIC-III

**DOI:** 10.1017/cts.2017.59

**Published:** 2018-05-10

**Authors:** Jianyin Shao, Ram Gouripeddi, Julio C. Facelli

## Abstract

OBJECTIVES/SPECIFIC AIMS: This poster presents a detailed characterization of the distribution of semantic concepts used in the text describing eligibility criteria of clinical trials reported to ClincalTrials.gov and patient notes from MIMIC-III. The final goal of this study is to find a minimal set of semantic concepts that can describe clinical trials and patients for efficient computational matching of clinical trial descriptions to potential participants at large scale. METHODS/STUDY POPULATION: We downloaded the free text describing the eligibility criteria of all clinical trials reported to ClinicalTrials.gov as of July 28, 2015, ~195,000 trials and ~2,000,000 clinical notes from MIMIC-III. Using MetaMap 2014 we extracted UMLS concepts (CUIs) from the collected text. We calculated the frequency of presence of the semantic concepts in the texts describing the clinical trials eligibility criteria and patient notes. RESULTS/ANTICIPATED RESULTS: The results show a classical power distribution, *Y*=2^10^ *X* ^(−2.043)^, *R* ^2^=0.9599, for clinical trial eligibility criteria and *Y*=5^13^ *X* ^(−2.684)^, *R* ^2^=0.9477 for MIMIC patient notes, where *Y* represents the number of documents in which a concept appears and *X* is the cardinal order the concept ordered from more to less frequent. From this distribution, it is possible to realize that from the over, 100,000 concepts in UMLS, there are only ~60,000 and 50,000 concepts that appear in less than 10 clinical trial eligibility descriptions and MIMIC-III patient clinical notes, respectively. This indicates that it would be possible to describe clinical trials and patient notes with a relatively small number of concepts, making the search space for matching patients to clinical trials a relatively small sub-space of the overall UMLS search space. DISCUSSION/SIGNIFICANCE OF IMPACT: Our results showing that the concepts used to describe clinical trial eligibility criteria and patient clinical notes follow a power distribution can lead to tractable computational approaches to automatically match patients to clinical trials at large scale by considerably reducing the search space. While automatic patient matching is not the panacea for improving clinical trial recruitment, better low cost computational preselection processes can allow the limited human resources assigned to patient recruitment to be redirected to the most promising targets for recruitment.

